# The Tenth Asia Pacific Bioinformatics Conference (APBC 2012)

**DOI:** 10.1186/1471-2164-13-S1-I1

**Published:** 2012-01-17

**Authors:** Yi-Ping Phoebe Chen, Peer Bork

**Affiliations:** 1 La Trobe University, Melbourne, Australia; 2 European Molecular Biology Laboratory, Heidelberg, Germany

## 

The Asia Pacific Bioinformatics Conference (APBC) is a leading conference in the Bioinformatics community and has grown rapidly since its inception in 2003. The goal of the annual conference series is to enable high quality interaction on bioinformatics research.

The past APBC conferences were held in:

1. APBC 2003 4-7 Feb 2003: Adelaide, Australia

2. APBC 2004 18-22 Jan 2004: Dunedin, New Zealand

3. APBC 2005 17-21 Jan 2005: Singapore

4. APBC 2006 13-16 Feb 2006: Taipei, Taiwan

5. APBC 2007 15-17 Jan 2007: Hong Kong

6. APBC 2008 14-17 Jan 2008: Kyoto, Japan

7. APBC 2009 13-16 Jan 2009: Beijing, China

8. APBC 2010 18-21 Jan 2010: Bangalore, India

9. APBC 2011 11-14 Jan 2011: Incheon, Korea

The Tenth Asia Pacific Bioinformatics Conference (APBC 2012) was held in Melbourne, Australia. The conference spanning the dates of the 17^th ^to the 19^th ^of January brought together more than 50 researchers, professionals, industry leaders and students from all over the globe. The participants came from institutions in the following 16 countries and regions (in alphabetical order): Australia, Canada, China, Germany, India, Netherlands, Japan, Poland, Portugal, Singapore, South Korea, Taiwan, Thailand, UK, USA and Vietnam. The conference program included 3 keynote speakers (Profs. Jenny Graves, Terry Speed, Peer Bork), 3 invited speakers (Profs. Steven Jones, Narayanaswamy Srinivasan, Robin Gasser), 42 selected talks, 3 tutorial sessions and 30 posters.

The titles of the keynote talks were:

• Jenny Graves, "Weird Animal Genomes and Sex"

• Peer Bork, "Networks in Biology: From functional associations of tiny molecules to entire species"

• Terry Speed, "Removing Unwanted Variation from Gene Expression Data"

The tutorial topics for APBC 2012 included: Analysis of Gene Expression and Proteomic Profiles based on Biological Networks (Limsoon Wong), Introduction to NGS informatics (Andrew Lonie), and Protein Inference: Concepts, Algorithms, and Software Tools (Zengyou He and Weichuan Yu).

The emphasis of APBC has been algorithmic development and innovation in Bioinformatics and this year that theme continued. Reflecting the ever-changing nature of Bioinformatics and its adaption to advances in technology, a strong emphasis was placed on post sequencing data mining. A wide range of topics in Bioinformatics were covered at the conference and were categorised as:

• Genome Evolution

• Regulatory Studies

• Gene Network Modelling

• Micro/RNA Modelling

• Database and Data Models

• Protein - Protein Interaction

• Protein Structural Prediction

• Algorithmic Optimisation and Evaluation

• Knowledge Reconstruction

• Knowledge Modelling

• Protein Drug Applications

• Protein Binding Studies

• Prediction Applications

In 2012, APBC received a substantial number of submissions from a diverse range of countries. Participants in the 2012 APBC came from every continent with the exception of Africa; however actual participants may represent geographies outside of their institutions. The APBC 2012 conference has received 129 submissions covering a wide range of topics in Bioinformatics.

The conference's scope was wide in order to adapt itself quickly to this progressive research field. The broad composition of the expert program committee was well equipped to handle the diversity of topics under the banner of Bioinformatics and Computational Biology. Of the 129 submitted papers, each paper was sent to three Program Committee members to review (102 papers received three peer reviews) and with an acceptance rate of 32.5%. We wish to thank and acknowledge the Program Committee members and their contributions.

The Program Committee members are:

• Tatsuya Akutsu, Kyoto University, Japan

• Masanori Arita, University of Tokyo, Japan

• Joel Bader, Johns Hopkins University, USA

• Vladimir Brusic, Dana-Farber Cancer Institute, USA

• Kun-Mao Chao, National Taiwan University, Taiwan

• Yi-Ping Phoebe Chen, La Trobe University, Australia

• Jake Yue Chen, Indiana U. School of Informatics, USA

• Francis Chin, The University of Hong Kong, Hong Kong

• Jung Kyoon Choi, KAIST, Korea

• In-Sun Chu, KRIBB, Korea

• Kyungsook Han, INHA University, Korea

• Wen-Lian Hsu, Academia Sinica, Taiwan

• Hsien-Da Huang, National Chiao Tung University, Taiwan

• Daniel Huson, University at Tubingen, Tubingen

• Jenn-Kang Hwang, National Chiao Tung University, Taiwan

• Ju Han Kim, Seoul National University, Korea

• Young Kim, KRIBB, Korea

• Hyunju Lee, GIST, Korea

• Sang Yup Lee, KAIST, Korea

• Wentian Li, Feinstein Institute for Medical Research, USA

• Jingchu Luo, Peking University, China

• Bin Ma, University of Waterloo, Canada

• Hiroshi Mamitsuka, Kyoto University, Japan

• Geoff McLachlan, University of Queensland, Australia

• Satoru Miyano, University of Tokyo, Japan

• Shinichi Morishita, University of Tokyo, Japan

• Kenta Nakai, University of Tokyo, Japan

• Mark Ragan, University of Queensland, Australia

• Naren Ramakrishan, Graduate Studies (bio), India

• Cenk Sahinalp, SFU, Canada

• Yasubumi Sakakibara, Keio University, Japan

• David Sankoff, University of Ottawa, Canada

• Thomas Schlitt, King's College London, United Kingdom

• Ramanathan Sowdhamini, NCBS, India

• Narayanaswamy Srinivasan, IISc, India

• Wing-Kin Sung, National University of Singapore, Singapore

• Alfonso Valencia, Centro Nacional de Biotechnologia, Spain

• Lusheng Wang, The City University of Hong Kong, Hong Kong

• Hong Yan, The City University of Hong Kong, Hong Kong

• Ueng Cheng Yang, National Yang Ming University, Taiwan

• S.M. Yiu, The University of Hong Kong, Hong Kong

• Louxin Zhang, National University of Singapore, Singapore

• Xuegong Zhang, Tsinghua University, China

• Hongyu Zhao, Yale School of Public Health, USA

• Jun Huan, University of Kansas, USA

We would also like to thank all local Organizing Committee members behind the scenes, especially Phoebe Chen, Scott Mann, Howard Lee, Subha Kalyaanamoorthy, for their hard work on the website, local arrangement and many other miscellaneous tasks. We also thank Publication & Web Chair Scott Mann, Ka Ho Chi and Howard Lee.

## Competing interests

The authors declare that they have no competing interests.

